# Investigation of serum lactate dehydrogenase levels in generalised anxiety disorder

**DOI:** 10.4102/sajpsychiatry.v32i0.2614

**Published:** 2026-07-20

**Authors:** Serkan Yazici, Ezgi Sıla Ahi Üstün

**Affiliations:** 1 Department of Psychiatry, Bursa City Hospital, Bursa, Turkey; 2 Department of Psychiatry, Mamak State Hospital, Ankara, Turkey

**Keywords:** lactate dehydrogenase, generalised anxiety disorder, lactate, energy metabolism, energy

## Abstract

**Background:**

Lactate dehydrogenase (LDH) plays a crucial role in glucose metabolism by catalysing the conversion of pyruvate to lactate and facilitating lactate utilisation. Given LDH’s central role in energy metabolism, it may serve as a promising biomarker for anxiety disorders.

**Aim:**

This study aimed to compare serum LDH levels between patients with generalised anxiety disorder (GAD) and healthy controls (HC) and to examine the associations between LDH levels and clinical features.

**Setting:**

Bursa City Hospital: A tertiary hospital in Bursa, Turkey.

**Methods:**

This study included 113 participants with GAD and 90 HC. Group comparisons were conducted using an independent samples *t*-test. Correlations between LDH levels and clinical variables were assessed using the Pearson rank correlation coefficient. To control for the impacts of possible confounding factors on LDH levels, an analysis of covariance (ANCOVA) was conducted.

**Results:**

Serum LDH levels were significantly lower in the GAD compared to the HC (157.7 ± 18.7 vs. 173.2 ± 23.3; *p*: <0.001, *t*: −5.249, d Cohen: 0.733). The ANCOVA analysis revealed that there was a statistically significant difference in adjusted LDH levels between the control and GAD groups (*p* < 0.001; mean difference: −15.607; 95% confidence interval [CI]: −21.180 to −9.975). Additionally, LDH levels were negatively and significantly correlated with HAM-A scores (*r* = −0.218, *p* = 0.021) and psychic subscale scores (*r* = −0.250, *p* = 0.008).

**Conclusion:**

These findings support the hypothesis that LDH may represent a potential biomarker and therapeutic target in the clinical management of anxiety.

**Contribution:**

This study is the first to report significantly reduced LDH levels in individuals with GAD, highlighting a potential link between altered lactate metabolism and anxiety pathogenesis.

## Introduction

The energy metabolism of the central nervous system plays a critical role in neuroplasticity, including neurogenesis, neural differentiation and neurotransmitter release.^1^ Given the well-established association between neuroplasticity and psychiatric disorders, it is unsurprising that recent years have witnessed an increasing number of studies investigating the disturbances in brain energy metabolism in the aetiology of psychiatric diseases.^2^ Alterations in brain energy metabolism have been particularly noted in mood disorders such as major depressive disorder (MDD) and bipolar disorder, psychotic disorders and neurodevelopmental disorders including autism spectrum disorders.^3,4,5^

The aetiology of anxiety disorders also involves multiple factors, including genetic, neurobiological, neurochemical and psychological components.^6^ As with most psychiatric disorders, the heterogeneous aetiology of anxiety disorders and the current reliance on clinical symptoms rather than objective biomarkers for diagnosis highlight the need to identify a broad range of biomarkers that could improve the understanding of etiological mechanisms and facilitate the development of patient-centred, targeted therapies.^7^ Numerous animal studies, which can serve as models for human research, have been conducted to elucidate the biological pathways potentially involved in the development of anxiety disorders.^8^ These studies suggest that alterations in brain energy metabolism, such as oxidative stress, mitochondrial energy pathway disruptions, impairments in glutamine metabolism and neurotransmission disturbances, may represent potential mechanisms.^9^ All these processes hold promise as candidate biomarkers for anxiety disorders.

Lactate, a metabolic intermediate produced during the breakdown of glucose or glycogen, plays a crucial role in brain energy metabolism. Additionally, lactate utilisation is involved in synaptic plasticity, memory processes and intercellular signalling.^10^ Lactate is converted into pyruvate by the enzyme lactate dehydrogenase (LDH), thus contributing to oxidative metabolism. The availability of lactate is essential for the maintenance of functional synapses. Reductions in LDH levels or enzymatic activity impair lactate metabolism and clearance, leading to lactate accumulation.^11^

Serum LDH is a non-specific enzyme present in nearly all body tissues, and its levels may be influenced by a range of systemic factors independent of psychiatric conditions.^12^ Elevated LDH levels are commonly associated with tissue damage or increased cell turnover, as seen in haemolytic anaemia, hepatic disease, renal disease, malignancy, pulmonary embolism and skeletal muscle injury, including that caused by vigorous physical activity.^13^ Conversely, reduced serum LDH levels have been reported in association with hypothyroidism, certain metabolic conditions and notably in the context of mood and psychotic disorders.^14^ These considerations highlight the non-specific nature of serum LDH and underscore the importance of controlling for potential confounders when interpreting LDH as a candidate biomarker in psychiatric research.

Lactate dehydrogenase is currently being evaluated as a potential biomarker for several neurodegenerative and psychiatric conditions, including Parkinson’s disease, Alzheimer’s disease, Huntington’s disease and MDD.^14^ There are also studies linking decreased cerebrospinal fluid (CSF) LDH levels with longer duration of untreated psychosis and increased symptom severity in patients with psychotic disorders.^15^

To date, no studies in the literature have specifically examined how LDH levels change in anxiety disorders. The primary objective of our study is to investigate LDH levels in patients with generalised anxiety disorder (GAD) and to explore the relationship between LDH levels and clinical parameters such as symptom severity, age of onset of illness and duration of untreated illness.

Our primary hypothesis is that LDH levels in patients with GAD will be altered compared to the healthy control (HC) group. The secondary hypothesis posits that there may be a correlation between LDH levels and parameters such as age of onset, duration of untreated illness and severity of clinical symptoms.

## Research methods and design

### Participants

This case–control study included 113 patients aged between 18 years and 65 years who presented to the outpatient psychiatry clinic between 01 October 2024 and 31 December 2024 and were diagnosed with GAD according to the DSM-5 criteria. Generalised anxiety disorder diagnoses were established by trained psychiatrists at the outpatient psychiatry clinic using DSM-5 criteria, independently of the research team. The control group comprised 90 individuals aged between 18 years and 65 years, recruited from the same hospital through pre-employment health screening or university enrolment examinations, with no history of psychiatric disorders. Exclusion criteria both for case and control group, included psychiatric medication use within the past three months, presence of comorbid psychiatric disorders, having a history of substance use in the last year or having been previously diagnosed with substance use disorder, alcohol use or abuse or dependence disorder, in the past lifetime, cardiovascular, hepatic, renal, pulmonary or skeletal muscle diseases, a history of malignancy and pregnancy or lactation. Patients were categorised as first-episode or recurrent GAD based on their prior psychiatric history. Those who had never previously received a formal diagnosis of GAD were classified as first episode. Patients who had a prior diagnosis of GAD, had achieved symptomatic remission following treatment and subsequently presented with a relapse requiring clinical attention were classified as recurrent. Patients’ medication abstinence history in the last 3 months was verified by checking through the national patient data system.

### Data collection instruments

#### Sociodemographic and clinical data form

Information regarding participants’ age, gender, smoking and alcohol use, age of onset of illness and duration of untreated illness was collected through patient interviews.

Alcohol consumption was assessed via structured patient interview, with participants reporting whether they consumed alcohol and, if so, their weekly intake in standard drink units. Participants with a diagnosis of alcohol use disorder or alcohol-related laboratory abnormalities, including elevated GGT, were excluded from the study. Alcohol consumption was subsequently included as a covariate in the ANCOVA analysis to control for its potential confounding influence on serum LDH levels.

#### Hamilton Anxiety Rating Scale

The Hamilton Anxiety Rating Scale (HAM-A) is a semi-structured instrument developed to assess the severity of anxiety disorders.^16^ It consists of 14 items; each scored between 0 and 3. The scale includes two subscales: psychic and somatic anxiety. The Turkish validity and reliability study of the scale was conducted by Yazıcı et al.^17^

#### Blood sampling

Blood samples were collected from the antecubital vein between 8:00 and 10:00, following a fasting period of 10–12 h. Serum samples were obtained by centrifugation at 3500 rpm for 5 min and were analysed within one hour. The determination of LDH levels was carried out via the NADH oxidation method using a fully automated clinical chemistry analyser. All analyses were processed at a single centre.

#### Statistical analysis

Statistical analyses were conducted using SPSS version 23.0 (Statistical Package for the Social Sciences for Windows, Version 23.0, IBM Corp., Armonk, New York, United States, 2015). A *p*-value of < 0.05 was considered statistically significant. Categorical variables (e.g. gender, smoking and alcohol use) were presented as frequencies and percentages, while continuous variables (e.g. age, HAM-A scores, LDH levels) were presented as mean, standard deviation, minimum, maximum and median values. Comparisons between groups were made using an independent samples t-test. Because of the normal distribution of numerical variables, relationships among numerical variables were evaluated using the ‘Pearson Correlation Coefficient’. In order to control for the effects of possible confounding factors on LDH levels, an ANCOVA (analysis of covariance) was conducted.

### Ethical considerations

All data were anonymised and stored securely in accordance with institutional protocols. No financial compensation was provided to participants. Ethical clearance to conduct this study was obtained from the Bursa City Hospital Human Research Ethics Committee (Ref. No. 2025-2/9).

## Results

### Sociodemographic and clinical characteristics

No significant differences were observed between the GAD and HC groups in terms of age, gender, smoking and alcohol consumption. However, LDH levels were found to be significantly lower in the GAD group compared to the HC group. The relevant data are presented in Table 1.

**TABLE 1:** Comparison of the healthy controls group and the generalised anxiety disorder group.

Variable	GAD (*n*: 113)			HC (*n*: 90)			Statistics
	Mean ± s.d.	*n*	%	Mean ± s.d.	*n*	%	
Age, year	38.0 ± 15.9	-	-	37.0 ± 12.0	-	-	*p*: 0.611
Gender, female	-	82	72.5	-	63	70	*p*: 0.688
Smoking	-	29	25.7	-	26	28.9	*p*: 0.608
Daily cigarette consumption	4.2 ± 4.6	-	-	3.8 ± 4.4	-	-	*p*: 0.717
Alcohol consumption	-	9	8.0	-	9	10.0	*p*: 0.612
Number of standard drinks per week	1.07 ± 4.6	-	-	1.02 ± 3.8	-	-	*p*: 0.808
LDH (U/L)	157.7 ± 18.7	-	-	173.2 ± 23.3	-	-	*p*: < 0.001, *t*: -5.249, *d*_*Cohen*_: 0.733 (95% CI: 0.448–1.017)
c*LDH (U/L)	157.6 ± 18.7	-	-	173.3 ± 23.4	-	-	*p*: < 0.001 MD: -15.607 (95% CI: -21.180, -9.975)
With first-episode GAD	-	51	45.1	-	-	-	-
With recurrent GAD	-	62	54.9	-	-	-	
HAM-A	22.8 ± 4.1	-	-	-	-	-	-
HAM-A- psychic	10.8 ± 2.5	-	-	-	-	-	-
HAM-A- somatic	11.9 ± 3.4	-	-	-	-	-	-
AoO	33.2 ± 14.1	-	-	-	-	-	-
DoUI (month)	5.3 ± 4.4	-	-	-	-	-	-

Note: One standard drink: The drinks containing 12 g – 14 g of ethyl alcohol (330 mL beer, 1 glass [140 mL] wine, 1 single [40 mL] vodka, whisky, gin or raki).

GAD, generalised anxiety disorder; HC, healthy control; LDH, lactate dehydrogenase; AoO, age of onset of illness; DoUI, duration of untreated illness; HAM-A, Hamilton Anxiety Rating Scale; s.d., standard deviation; MD, mean difference; CI, confidence interval; c*, corrected with ANCOVA.

#### Comparative analysis of serum lactate dehydrogenase levels in subgroups and relationship between lactate dehydrogenase levels and potential confounding variables

The relationship between LDH levels and potential confounding variables such as age, gender, cigarette and alcohol consumption was examined. The analysis revealed no statistically significant association between LDH and age, gender or the number of cigarettes consumed per day (respectively, *p*: 0.317, *p*: 0.763, *p*: 0.153). However, a significant relationship was observed between LDH levels and the amount of weekly alcohol consumed (*p*: <0.001, *r*: 0.264). Lactate dehydrogenase comparisons between subgroups are presented in Table 2. Following the ANCOVA analysis, a statistically significant difference in adjusted LDH levels between the control and GAD groups was observed (*p* < 0.001; mean difference: −15.607; 95% confidence interval [CI]: –21.180 to –9.975).

**TABLE 2:** Lactate dehydrogenase comparisons between subgroups.

Variable	Mean ± s.d.	Statistics
**Gender**		*p*: 0.111
Male	165.3 ± 23.1	-
Female	162.7 ± 19.8	-
**Smoking**		*p*: 0.202
Yes	166.6 ± 24.1	-
No	164.9 ± 21.6	-
**Alcohol consumption**		*p*: 0.012 *t*: -2.542
Yes	177.1 ± 29.6	-
No	163.3 ± 21.1	-
**Episodes**		*p*: 0.707
First episode	158.4 ± 19.2	-
Recurrent	157.1 ± 18.4	-

s.d., standard deviation.

#### Comparative analysis of serum lactate dehydrogenase levels in different clinical subgroups of generalised anxiety disorder

**FIGURE 1:**
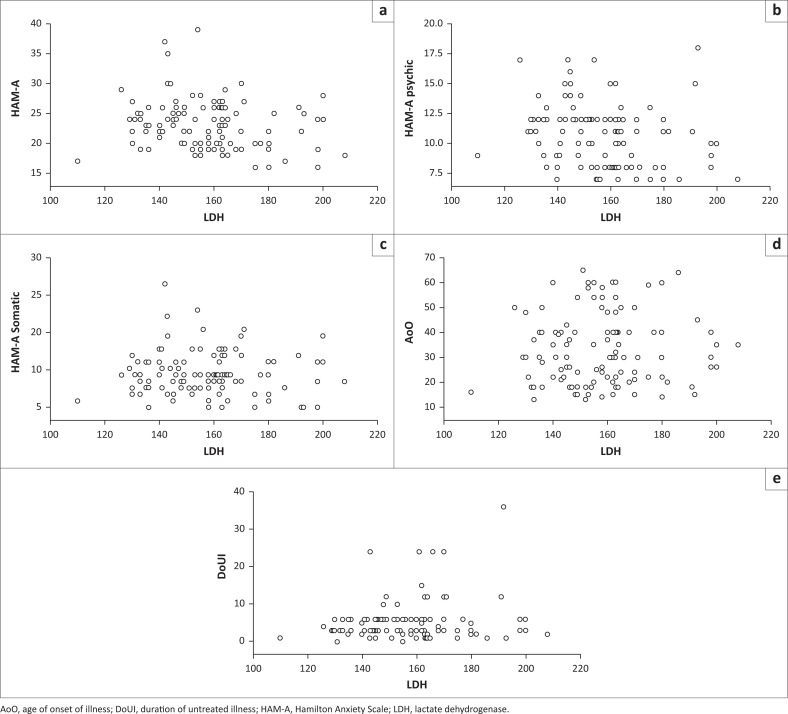
Correlation analysis of serum lactate dehydrogenase with (a) Hamilton Anxiety Rating Scale(HAM-A) total score; (b) HAM-A psychic subscale score, (c) HAM-A somatic subscale score; (d) age of onset of illness and (e) duration of untreated illness.

In the GAD group, a significant negative correlation was found between LDH levels and both the HAM-A total score and the psychic subscale score, whereas no significant relationship was observed between LDH levels and the somatic subscale score (*r* = −0.218, *p* = 0.021; *r* = −0.250, *p* = 0.008; *r* = 0.079, *p* = 0.405, respectively). No significant correlation was detected between LDH levels and either age of onset or duration of untreated illness (*r* = 0.001, *p* = 0.989; *r* = −0.178, *p* = 0.060, respectively). Additionally, no significant difference in LDH levels was found between subgroups of patients experiencing their first episode and those with recurrent episodes (157.1 ± 18.4 vs. 158.4 ± 19.2, *p* = 0.707). The relevant analyses are presented in Table 3 and Figure 1.

**TABLE 3:** Correlation between serum lactate dehydrogenase and clinical data.

Variable	LDH	
	*r*	*p*-value
HAM-A	−0.218	0.021
HAM-A- psychic	−0.250	0.008
HAM-A- somatic	−0.079	0.405
AoO	0.001	0.989
DoUI	−0.178	0.060

AoO, age of onset of illness; DoUI, duration of untreated illness; GAD, generalised anxiety disorder; HAM-A, Hamilton Anxiety Rating Scale; HC, healthy control; LDH, lactate dehydrogenase.

## Discussion

In our study, we found that LDH levels were significantly lower in the GAD group compared to the HC group. Moreover, we identified a correlation between the decrease in LDH levels and the severity of symptoms. However, no association was found between LDH levels and the age of onset or the duration of untreated illness. To our knowledge, this is the first study to investigate LDH levels in anxiety disorders.

The mean age of onset in our sample (33.2 ± 14.1 years) is higher than that reported in many community-based epidemiological studies, which often describe onset in childhood, adolescence or early adulthood.^18^ However, it is well established that GAD demonstrates considerable variability in age of onset across the lifespan,^19,20^ and treatment-seeking populations recruited from tertiary outpatient settings tend to present later than those identified in community samples.^21^ The wide standard deviation observed in our sample further reflects this heterogeneity. These factors may account for the later mean age of onset in our cohort and should be considered when interpreting the generalisability of our findings.

Our findings suggest that LDH may play a role in the pathogenesis of anxiety disorders. Alterations in lactate metabolism, which is closely associated with LDH activity, may represent a potential underlying mechanism.

In recent years, lactate has been increasingly recognised not merely as a byproduct of energy metabolism but as a significant glial-neuronal signalling molecule.^22^ Particularly, lactate derived from glial cells is closely associated with cognitive functions. Studies have shown that lactate utilisation can exert antidepressant effects by promoting adult hippocampal neurogenesis, whereas excessive lactate accumulation in the hippocampus may suppress this neurogenesis.^23^ The growing body of literature indicating significant changes in cerebral lactate levels across various psychiatric disorders suggests that lactate could be a potential target for therapeutic intervention in mental disorders.^24^

The relationship between lactate metabolism and anxiety-related behaviours is well established. In a study by Pitts and McClure, intravenous lactate infusion was found to increase anxiety symptoms in both patients with anxiety disorders and controls.^25^ While subsequent studies have reported similar findings, recent research has also highlighted the role of dietary and gut microbiome-derived lactate in the development of anxiety.^24,26,27^ Elevated lactate levels may exacerbate anxiety symptoms through mechanisms such as reduced alveolar carbon dioxide concentration and γ-aminobutyric acid (GABA) dysfunction in the dorsomedial hypothalamus because of increased lactate in the CSF.^28,29^

A decrease in LDH levels may disrupt lactate metabolism, leading to lactate accumulation. Considering the association between elevated lactate levels and anxiety behaviours, reduced LDH activity might contribute to the emergence of anxiety symptoms. In line with this, our study found significantly lower LDH levels in the patient group compared to controls. Furthermore, the observed inverse correlation between LDH levels and symptom severity is consistent with the literature, indicating that lactate infusion exacerbates anxiety symptoms.

Although the absolute difference in serum LDH between groups (15.6 U/L) may appear modest relative to the broad normal laboratory reference range, the associated effect size was moderate (Cohen’s *d* = 0.733), suggesting a meaningful biological signal. Furthermore, experimental evidence indicates that even relatively small reductions in LDH activity can impair lactate clearance and contribute to lactate accumulation,^14^ which has been independently linked to the exacerbation of anxiety symptoms.^25^ We therefore propose that the biological significance of this difference should not be assessed solely against standard laboratory thresholds but interpreted within the context of lactate metabolism and its established role in anxiety pathophysiology.^24^ Future studies employing repeated measures and larger sample sizes will be necessary to define clinically actionable LDH thresholds in anxiety disorders.

Given the practicality and low cost of measuring LDH, serum LDH could potentially serve as a useful biomarker in anxiety disorders. We believe that the findings of our study may be easily translatable to clinical practice and thus hold significant value. Nonetheless, we found no correlation between LDH levels and several clinical variables that we hypothesised might influence LDH activity. Further studies with larger sample sizes are warranted to re-evaluate these clinical variables.

We acknowledge several limitations in our study. Initially, we only assessed serum LDH levels and could not evaluate the correlation between serum and CSF LDH levels; therefore, the interpretation of peripheral serum LDH as a surrogate marker of central metabolic processes should be approached with caution, as peripheral LDH levels do not directly reflect brain LDH activity.^30^ Additionally, although blood samples were collected under standardised fasting conditions in the early morning, formal assessment of participants’ recent physical activity levels was not performed, which may represent a potential source of variability in serum LDH measurements.^12^ In addition, the lack of analysis regarding LDH isoenzymes represents another limitation of our study. Furthermore, diagnoses were established by a trained psychiatrist based on DSM-5 criteria through clinical interview; however, a formal structured diagnostic instrument such as the Structured Clinical Interview for DSM-5 (SCID-5) was not employed, which may limit the standardisation of diagnostic assessment. The exclusion of comorbid psychiatric disorders, while necessary to preserve internal validity, limits the generalisability of our findings to clinical populations with GAD and co-occurring conditions, which represent the majority of patients encountered in routine psychiatric practice.

Several directions for future research emerge from the findings of the present study. Firstly, longitudinal studies are needed to examine whether changes in serum LDH levels over time correspond to fluctuations in anxiety symptom severity, treatment response or clinical remission, which would help clarify the potential utility of LDH as a dynamic biomarker in GAD. Secondly, studies simultaneously measuring both peripheral serum and CSF LDH levels would allow a more direct assessment of the relationship between central and peripheral lactate metabolism in anxiety disorders. Thirdly, analysis of LDH isoenzyme profiles may provide greater specificity regarding the tissue origin of observed LDH alterations. Fourthly, future research should include patients with comorbid psychiatric conditions to determine whether the observed LDH reductions are specific to GAD or represent a transdiagnostic biomarker of anxiety-related psychopathology. Fifthly, multicentre studies with larger and more diverse samples are warranted to confirm the reproducibility and generalisability of these findings across different clinical and cultural settings.

## Conclusion

The findings of our study are consistent with the growing body of literature suggesting a potential link between impaired brain energy metabolism and anxiety disorders. There is a clear need for objective biomarkers to deepen our understanding of the underlying causes of psychiatric disorders. We believe that our study contributes to this rising awareness and may serve as a precursor to future multicentre studies with larger sample sizes.
